# An Approach to Precise Nitrogen Management Using Hand-Held Crop Sensor Measurements and Winter Wheat Yield Mapping in a Mediterranean Environment

**DOI:** 10.3390/s150305504

**Published:** 2015-03-06

**Authors:** Lucía Quebrajo, Manuel Pérez-Ruiz, Antonio Rodriguez-Lizana, Juan Agüera

**Affiliations:** 1 Aerospace Engineering and Fluids Mechanics Department, University of Seville, Ctra. Sevilla-Utrera km. 1, Seville 41013, Spain; E-Mails: lquebrajo@us.es (L.Q.); arodriguez2@us.es (A.R.-L.); 2 Rural Engineering Department, University of Cordoba, Córdoba 14071, Spain; E-Mail: jaguera@uco.es

**Keywords:** NDVI, yield estimation, winter wheat

## Abstract

Regardless of the crop production system, nutrients inputs must be controlled at or below a certain economic threshold to achieve an acceptable level of profitability. The use of management zones and variable-rate fertilizer applications is gaining popularity in precision agriculture. Many researchers have evaluated the application of final yield maps and geo-referenced geophysical measurements (e.g., apparent soil electrical conductivity-ECa) as a method of establishing relatively homogeneous management zones within the same plot. Yield estimation models based on crop conditions at certain growth stages, soil nutrient statuses, agronomic factors, moisture statuses, and weed/pest pressures are a primary goal in precision agriculture. This study attempted to achieve the following objectives: (1) to investigate the potential for predicting winter wheat yields using vegetation measurements (the Normalized Difference Vegetation Index—NDVI) at the beginning of the season, thereby allowing for a yield response to nitrogen (N) fertilizer; and (2) evaluate the feasibility of using inexpensive optical sensor measurements in a Mediterranean environment. A field experiment was conducted in two commercial wheat fields near Seville, in southwestern Spain. Yield data were collected at harvest using a yield monitoring system (RDS Ceres II-volumetric meter) installed on a combine. Wheat yield and NDVI values of 3498 ± 481 kg ha^−1^ and 0.67 ± 0.04 nm nm^−1^ (field 1) and 3221 ± 531 kg ha^−1^ and 0.68 ± 0.05 nm nm^−1^ (field 2) were obtained. In both fields, the yield and NDVI exhibited a strong Pearson correlation, with r_xy_ = 0.64 and *p* < 10^−4^ in field 1 and r_xy_ = 0.78 and *p* < 10^−4^ in field 2. The preliminary results indicate that hand-held crop sensor-based N management can be applied to wheat production in Spain and has the potential to increase agronomic N-use efficiency on a long-term basis.

## 1. Introduction

The goal of site-specific management practices is to enable more efficient use of fertilizers, pesticides, fuel, management and labor inputs. Most farming systems use spatial variability information related to crop status and soil characteristics to implement innovative management strategies to achieve a site-specific scenario. This new method of implementing agriculture is being bolstered by emerging cost-effective remote sensing techniques. Field crops must receive appropriate rates of nitrogen (N) fertilizer to achieve optimal yields; both underfertilization and overfertilization can negatively affect the desired growth pattern of plants and reduce yields. Furthermore, repeated machinery passes for N applications increase driving distances, require more time, increase soil compaction, consume more farming inputs and increase the environmental load [1].

Andalucia, in southern Spain, serves as an example of high agricultural value and represents 62% of the area (197,826.00 ha) used for and more than 80% of the national production of winter wheat, with an average yield of 3.11 t ha^−1^ (MAGRAMA [2], advancing production and area, July 2014). Using the average N fertilization application rate of 120 kg ha^−1^ per year and a price of 8–9 € ha^−1^ for application by a contractor company (two passes per fertilization) at 110 € t^−1^ of urea with 46% N amounts to a cost of 46.5 € ha^−1^ cost per year (fertilizer plus application). As much as 20% of inputs can be saved with the use of precision farming techniques and variable rate fertilizer application using proper machinery and precision application in areas with good yields and reduced inputs in areas with low yields (in which the lower yield may be due to soil limitations rather than insufficient N fertilization). 

In this case, the cost would be 37.2 € ha^−1^ per year for the same yield at the end of the season. Andalucia could save approximately 1.8 M € using precision agricultural techniques for N application. Raun and Johnson [3] reported that conventional N management strategies in world cereal production systems have resulted in a lower percentage of applied fertilizer N being recovered in the aboveground crop biomass during the growing season; they estimate that an average of only 33% of fertilizer N is recovered. Although it is impossible to achieve 100% efficiency of N fertilizer use in any production system worldwide, the use of large amounts of N fertilizer suggests that there is a significant opportunity for reducing N losses associated with conventional practices.

Detailed knowledge of the relationship between applied fertilizer and crop yield in a zone under given soil conditions may be obtained through numerical approximations. Crop production models can be characterized as empirical and mechanistic (process-oriented) models. Empirical models directly employ a relationship between model variables and model outputs without requiring a description of fundamental (physical) processes. These models are usually site specific. Mechanistic models are often more complex because they describe known physical and biological processes in crops and soils. There are several models, such as the NDICEA model [4] that explore the relationship between applied N levels and their relationship to crop yield. The Quantitative Evaluation of the Fertility of Tropical Soils (QUEFTS) model, which is based on both theoretical and empirical relationships. 

Janssen *et al.* [5], showed that N applications improve crop yields [6], which is in agreement with the empirical results obtained by Wild [7] and González-Fernández [8] for wheat under various soil management systems. The use of these and other models may be of interest from an agronomic and environmental perspective because they improve on the traditional method of trial and error, which is more time consuming, and contribute to better decision making by farmers, thus resulting in savings in terms of fertilizer applied to the crop.

Remote sensing information is an integrated manifestation of the effects of on-field factors, such as soil texture, pH, biological and chemical factors, and of external factors, such as farming practices and weather conditions, on crop growth; therefore, remote sensing can have a substantial impact in improving yield estimation [9,10]. Indirect sensing methods for measuring the ratio of vegetation indices (VIs) have been widely used to quantify crop variables such as yield and biomass; such methods may be available at different levels, depending on the type of platform that carries the imaging sensor, *i.e.*, satellite [11], aerial [12] or ground [13] vehicles. Farmers who can distribute the additional cost of improved management over a large operation can better absorb the high costs of satellite images (large-scale observation). Platforms that assess crop vigor cannot typically be used by small and mid-size agricultural operations because their costs can be very high and thus unprofitable for small-scale crops. The intrinsic drawback associated with satellite observations is the temporal frequency of satellite data. The degree of correspondence between the temporal frequencies of passive satellite remote sensor data collections and varying processes or crop statuses can significantly impact the accuracy of change detection monitoring efforts. Furthermore, the presence of clouds can reduce the number of opportunities for satellite data collection [14].

Small, unmanned aircraft systems and ground-based remote sensing hold great promise for small and mid-sized farmers because of their rapid development and decreasing costs [15,16]. For ground-based images or the determination of VIs, an optical sensor with a computer recording sensor output may be mounted on the implement/tractor, or it may be used in survey mode (by hand); the global navigation satellite system (GNSS) field position is provided in both situations. The use of hand-held sensors can provide similar results at a much lower cost, which would make crops profitable and precision nutrient management (e.g., N) possible on small scales. 

The spectral reflectance determined from image data has been used to calculate various Vis, such as the normalized difference vegetation index (NDVI), which is calculated by dividing the difference between the reflectances of the NIR and red bands by the sum of the reflectances of the NIR and red bands, *i.e.*, NDVI = (NIR − Red)/(NIR + Red). A variety of VIs, including reflectance band ratios and individual band reflectance, have also been used for crop management and yield prediction [17,18]. In terms of the N content, Tarpley *et al.* [19] found that reflectance ratios calculated by dividing cotton leaf reflectances at 700 or 760 nm by a higher wavelength reflectance (755 to 920 nm) can provide accurate predictions of N concentrations. Furthermore, Shanahan *et al.* [20] and Zarco-Tejada *et al.* [21] reported that N-status remote sensing is feasible for cereals and cotton, respectively.

The objectives of this work can be summarized as follows:

To measure the NDVI of a winter wheat field under commercial management using a hand-held active remote sensing device and to determine the real wheat N content in collected leaf samples using laboratory analysis. The yield information (field level) will be obtained using a commercially available grain yield monitor.
To determine the extent of spatial variability and co-variation between the wheat yield, N content and NDVI in two conventionally managed commercial fields used for wheat production.

## 2. Materials and Methods

This study was performed in two experimental plots (plot 1: 1.60 ha and plot 2: 1.21 ha) located in the western half of Andalucia (Southern Spain) in an area called the Countryside of Seville (Latitude: 37.4555477 N, Longitude: 5.4336677 W). The climate in this area is generally considered to be Mediterranean (summers that are dry and hot, low rainfall and strong evaporation demand). Typical crops in this area are arable crops and olive groves; therefore, we will study winter wheat because it is one of the most widespread crops in this area. In this case, the variety of wheat being grown is called COREL.

The soil is a vertisol, chromic haploxerert (Soil Survey Staff, 2014), formed on a Miocene marl. Clay is the predominant soil, with 64% in the 0–0.25 m layer, while sand makes up only 8% of the soil. The high carbonate content, 6.80%, results in a high pH value of 8.3. The available P content is 17.9 mg kg^−1^, and the oxidizable organic matter is 1.65%. The ratio C/N is 9.82.

A typical wheat crop management scheme in this area consists of the following steps (and dates): basal fertilization on 22 December 2013; sowing on 24 December 2013; nitrogen fertilizer application on 3 February 2014; Herbicide treatment on 6 March 2014; fungicide treatment on 9 April 2014 and harvest on 5 June 2014. 

### 2.1. Hand-Held Optical Sensor and GNSS Control Unit

A commercial portable hand-held device (GreenSeeker^®^, Trimble Navigation Ltd., Sunnyvale, CA, USA) was used to measure the spectral NDVI of field-grown wheat leaves. The optical sensor emits a brief burst of radiation from red (Red; 660 ± 15 nm) and near-infrared (NIR; 770 ± 15 nm) light-emitting diodes (LEDs) to collect reflectance data that are independent of the solar conditions. The specific optical sensor was chosen because of its affordability and easy handling by field technicians and farmers.

The device measures the NDVI with a push of a button. The NDVI values range from 0.00 to 0.99. The liquid crystal display (LCD) provides the NDVI reading, with higher values indicating a more vigorous and healthy crop [22]. Following the manufacturer recommendations, measurements were taken at a vertical viewing angle from a distance of 0.5–0.6 m above the crop to ensure accurate readings. The sensor’s field of view is an oval which, and it widens as the height of the sensor above the ground increases. A completely randomized design of 30 field-testing zones was used to perform the NDVI measurements.

**Figure 1:**
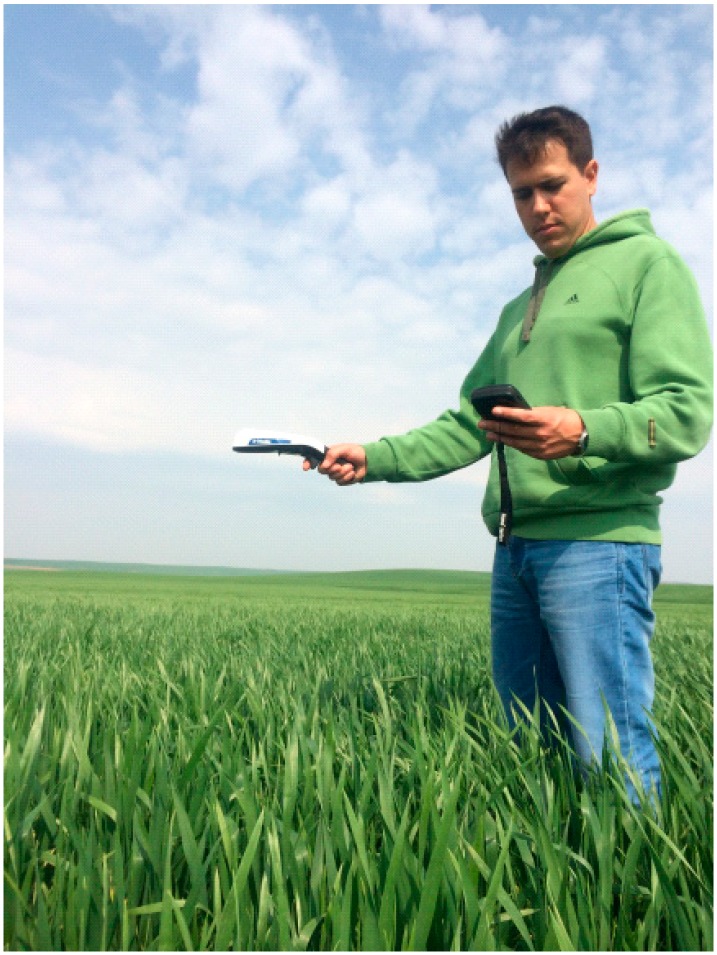
Hand-held NDVI sampling system in the experimental field.

A field computer (Juno 5D, Trimble Navigation Ltd., Sunnyvale, CA, USA) was used to record the location of the technician at all times. This computer recorded the location, time, date, number of satellites and NDVI reading in the internal memory. The Juno 5D differential GNSS receiver utilizes a technology that combines a GNSS receiver, a differential beacon receiver and differential satellite receiver in the same housing. The satellite differential receiver uses EGNOS (European Geostationary Navigation Overlay Service) correction signals from any source that transmits the signals in Radio Technical Commission for Maritime Services (RTCM) format (Figure 1). 

### 2.2. Yield Monitoring System

An RDS Ceres II yield monitoring system and an RDS GPS 16 receiver were installed on a Claas-Mega 216 combine harvester to estimate and record the yield and positional data, respectively. The yield monitor measured the moisture and yield data, whereas the GNSS receiver used EGNOS to obtain location data to within 3 m. The instantaneous yield, moisture, and GNSS data were simultaneously logged at two-second intervals onto a Secure Digital (SD) card installed on the yield monitor. The combine had an effective cutting width of 6 m and traveled at an average speed of 4.5 km/h. Therefore, approximately one sample was collected from a 15-m^2^ area.

**Figure 2:**
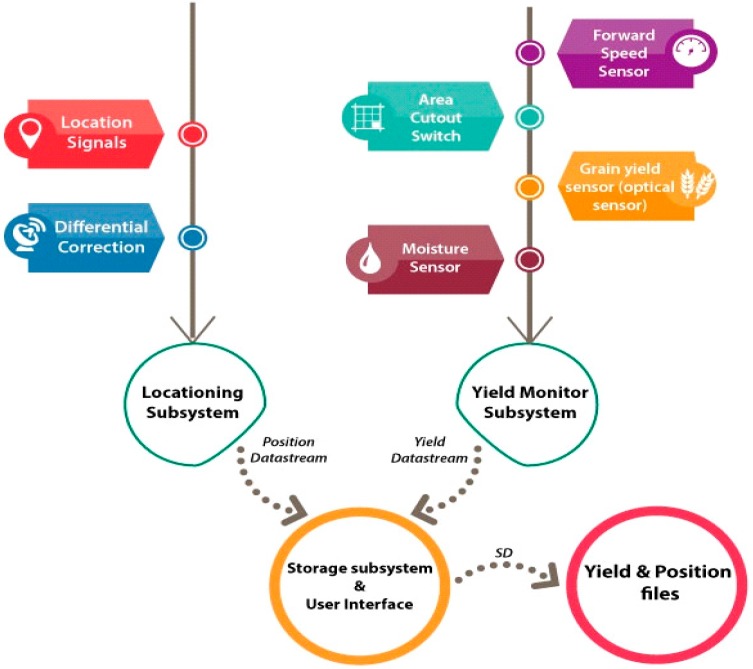
A block diagram of the yield monitoring system components.

The optical sensor used to measure the grain yield was fitted onto the upper part of the clean grain conveyor just before the grain was dropped into the grain tank. An infrared light beam was transmitted across the elevator paddles from one side to the other. A receiver detected when the light beam was blocked and when it was clear. As each paddle passed the sensor, the beam was blocked. The more grain there was on the paddle, the longer the beam was blocked. The transmitter and receiver, together with their lenses and lens holders, were each secured to a hinged mounting bracket that was attached to the elevator housing. The sensor operation was indicated by an LED on the end of each sensor. Figure 2 presents the linkages and setup of the sensor.

### 2.3. Leaf N Test and Field Experiments

To determine the real wheat N content, leaf sample collection and laboratory analyses were conducted using the protocol of Mills and Jones [23]. The leaves were collected and specially handled to ensure that no loss in dry weight from decomposition occurred because such loss would significantly impact the plant analysis results. All the leaf samples were geo-referenced using a Juno 5D differential GNSS receiver; placed in open, clean paper bags; and kept in a cool (5–7 °C), environment during shipment to the laboratory to prevent N volatilization.

At the time of leaf sampling, ear emergence had not occurred; therefore, in accordance with Mills and Jones [23], between the 4th and 5th leaf, the highest, *i.e.*, the youngest, leaf was taken because this leaf best defines the nutritional status of the crop. For each location sample, approximately 50 leaves (one per wheat plant) and 20 samples per field (two fields) were taken. Each field was homogeneous in terms of the soil and crop management. The N content was determined at the CITIUS laboratory (University of Seville, Seville, Spain) using the LECO CNS-2000 instrument (LECO Corp., St. Joseph, MI, USA). 

Field tests were performed during the spring of 2014 at a commercial wheat field (Latitude: 37.4555477 N, Longitude: 5.4336677 W). On 25 March 2014 (at the early growth), the NDVI measurements and leaf sample collection were obtained from two fields (1.60 ha and 1.21 ha). The NDVI values for each 350-m grid cell and the N percentage of leaves in 700-m grid cells within the study area were determined. On 2 June 2014, the harvest was conducted with the yield monitor system in place. The standard harvest procedures were slightly modified for calibration purposes to obtain accurate and completely site-specific yield information using the monitoring system.

### 2.4. AgGIS Software and Data Analysis

The Agricultural Geographic Information System (Farm Works TM, Trimble Navigation Ltd., Sunnyvale, CA, USA) software package was used to facilitate the development of plans and maps that highlighted different features for the design of applications in the study area and to incorporate all the field data. The recorded data from the Juno 5D differential GNSS receiver were imported into the Farm Works TM software package to create different maps and applications. 

The inverse distance weighting (IDW) method implemented in the AgGIS software package was used for interpolation. It was assumed that spatial distribution variable, Z, decreased linearly (*n* = 1) with distance. The size of the neighborhood and number of neighbors are also relevant to the accuracy of the results (*N* = 12).

(1) $$Zo\;=\frac{\sum_{i=1}^{N}z_{i}.d_{i}^{-n}}{\sum_{i=1}^{N}d_{i}^{-n}}$$

The variables in the above equation are the following:

Zo = the estimated value of the variable z at point i,
z_i_ = the sample value at point i,
d_i_ = the distance from one sample point to an estimated point,
n = the coefficient that determines the weight based on a distance, and
N = the total number of predictions for each validation case.

Once the data from all the trials were obtained, it was important to perform a statistical analysis before drawing conclusions using the R software package [24]. R is a free software package for statistical computing and visualization that is distributed under the terms of the Free Software Foundation’s GNU General Public License in source code form. This software is valued for its large variety of statistical methods and visualization capabilities.

In the statistical analysis, Pearson’s coefficient, which is an index that measures the degree of covariance between linearly related variables and which ranges between −1 and +1, was obtained, and relationships between NDVI and wheat yield and NDVI and leaf N content were determined using the method of ordinary least squares.

## 3. Results and Discussion

In Andalucía, Spain, the spatial statistical analysis of yield-monitored data has become relatively common for hand-harvested crops. This region is characterized by medium-large fields with homogeneous crops, and yield monitoring is often considered to be the main entry-level technology for use in precision agriculture. Spatial differences in wheat yields reflect differences in soil conditions that can, for instance, be repaired with fertilizer. These conditions are structural and are often reflected in the same patterns in the yields.

### 3.1. Yield Sensor Calibration and Yield Measurement

Achieving accurate wheat yield measurements is challenging, and calibration effects the yield measurements. RDS Ceres operates with a light barrier in the upper part of the feed-flow side of the clean grain elevator. The grain piles on the elevator paddles interrupt the light beam. The zero tare value is obtained from the darkening rate when the elevator is running empty. Each day, taring was performed for both the combined yield sensor and moisture sensor. The new calibration factor used in the yield monitoring in this study was calculated using the following, as proposed by Demmel *et al.* [25]:

(2) $$\text{Calibration}\;\text{factor}\;\text{yield}=\frac{\left(\text{Existing}\;\text{factor}\;\times \;\text{Weight}\;\text{measured}\right)}{\text{Weight}\;\text{from}\;\text{the}\;\text{console}}$$

The mean relative error represents a measure of the calibration quality. It should ideally be zero, or at least near zero. In this study, the (average) relative calibration error was −3.1%, with a standard deviation of 4.2% (5 repetitions). This error was primarily due to the different specific weights of the wheat grains from different parts of the field. These values are very similar to those obtained by Demmel *et al.* [26], with a mean of −0.14% and a standard deviation of 3.43%. Calibration is an important step for verifying the yield sensor output.

### 3.2. Relationship between the Wheat Yield and NDVI

**Figure 3:**
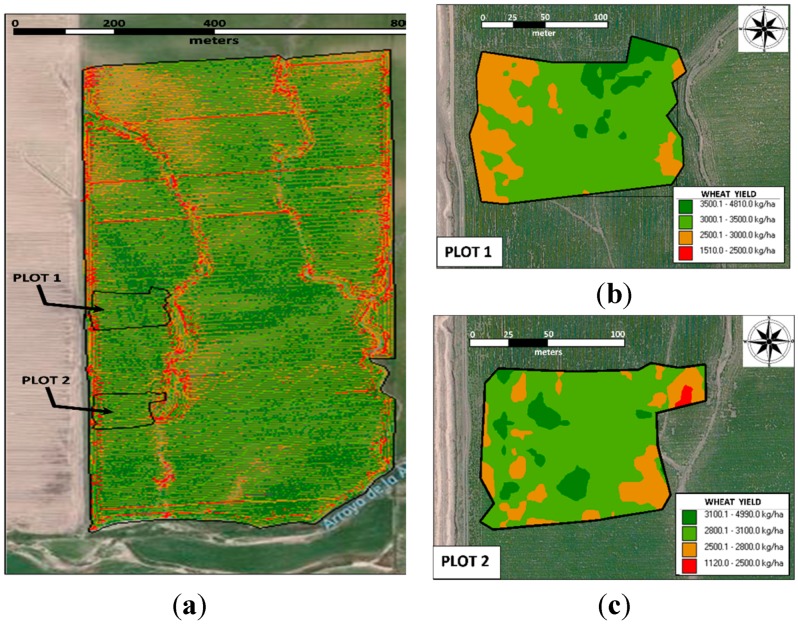
(**a**) Map of the wheat yield and two NDVI sampling sites; (**b**) plot 1 and (**c**) plot 2.

**Figure 4:**
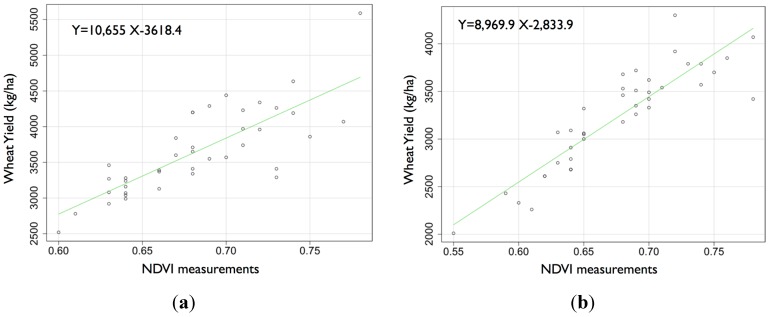
Relationship between the NDVI measurements and wheat yield (**a**) for field 1 and (**b**) for field 2.

**Figure 5:**
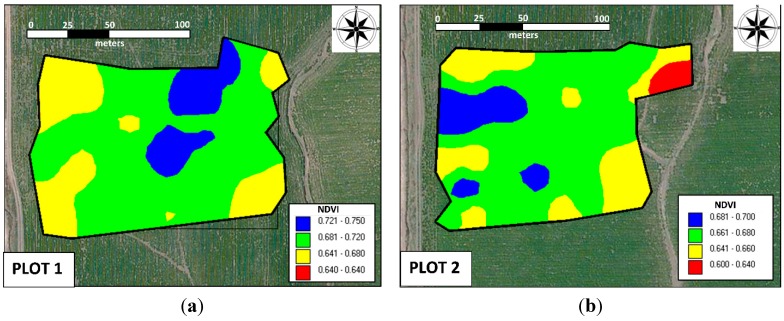
Spatial distribution of NDVI in both fields: (**a**) field 1 and (**b**) field 2.

The values of the wheat yield and NDVI for both fields were, respectively 3498 ± 481 kg ha^−1^ and 0.67 ± 0.04 nm nm^−1^ (field 1) and 3221 ± 531 kg ha^−1^ and 0.68 ± 0.05 nm nm^−1^ (field 2) (Figure 3). In both fields, the yield and NDVI exhibited a strong Pearson’s correlation, with r*_xy_* = 0.64 and *p* < 10^−4^ for field 1 and r*_xy_* = 0.78 and *p* < 10^−4^ for field 2 (Figure 4). According to the categorizations by Dancey and Reidy [27], these results denote a strong correlation between the yield and NDVI for both fields. The strong correlation suggests that NDVI measurements can be used for delineating management zones across a field, and such reliable and precise fertilizer (N) application has the potential to increase capacities in our agricultural environment. The visual similarity of the spatial distributions of the NDVI (Figure 5) and wheat yield confirms the close relationship between the two factors.

### 3.3. Relationship between Optical Sensor Measurements and Percentage of leaf N Content

**Figure 6:**
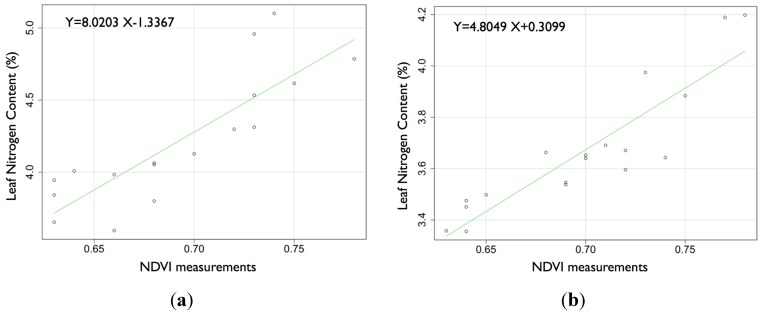
Relationship between the NDVI measurements and leaf N content (a) for field 1 and (**b**) for field 2.

Two field experiments were designed to determine the relationship between optical sensor measurement and the percentage of leaf N content. The measurements included 35 wheat leaf N content values (*n* = 17 for field 1 and *n* = 18 for field 2). The leaf N (%) values were 4.2% ± 0.44% (field 1) and 3.6% ± 0.24% (field 2). Figure 6 indicates a strong linear correlation between the N percentage content and NDVI measurements, which results in the following values: r*_xy_* = 0.71 and *p* < 10^−4^ for field 1 and r*_xy_* = 0.89 and *p* < 10^−4^ for field 2.

### 3.4. Potential Value of Variable Rate N Application

A potential application of the NDVI sensor is to perform variable-rate N management for winter wheat, as shown in Figure 6. Currently, the method for determining the N (e.g., urea) requirements of wheat is to use the N-balance method; in some cases, estimation of the N application needed per hectare is based on the technician’s and producer’s experience.

In the same campaign and field, previous work evaluated N response tests with total N rates of 55, 150, 190, 225 and 250 kg ha^−1^ to determine the relationship between the NDVI measurements and total N. This relationship revealed a clear exponential regression in the measurements (r*_xy_* = 0.99 and *p* < 10^−4^). The exponential regression was as follows

*y* = 15.573*e*^4.2795*x*^ (3)

where the variables are as follows:

*y* = Total N (kg ha^−1^)
*x* = NDVI sensor measurements

**Figure 7:**
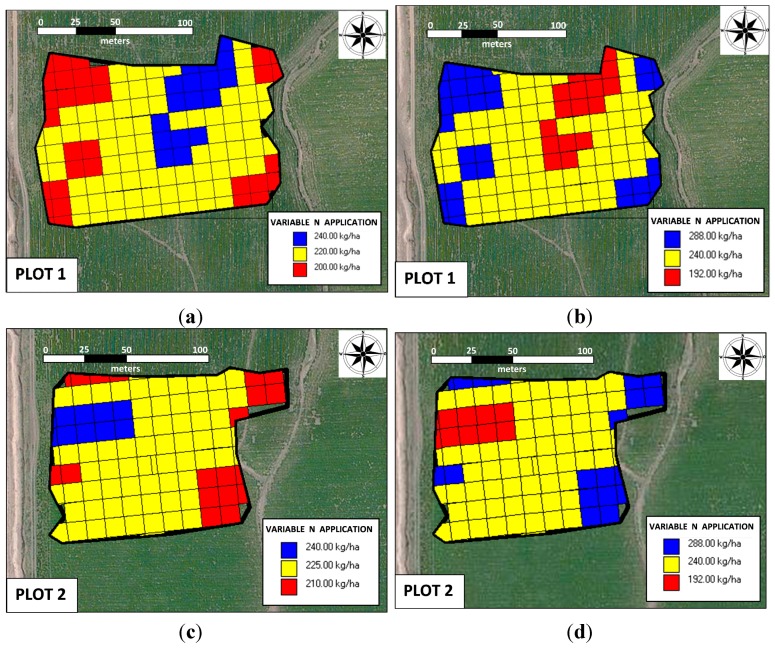
Site-specific precise nitrogen management units for two fields: (**b**) and (**d**) conservative application, and (**a**) and (**c**) risky application

The use of optical sensors resulted in NDVI measurements that ranged between 0.6 and 0.78 in both fields. This information, along with the standard spatial interpolation method of IDW, allowed for variable-rate N prescription maps to be generated [28]. Figure 7 exhibits a preliminary approach to applying N at variable rates in fields to meet the predicted wheat N needs; however, it is important to note that the spatial patterns of optimum N rates for the same field can vary from year to year. According by Pedroso *et al.* [29], for a field with precision farming, segmentation methods exist, and interest in such methods among researchers is increasing.

Figure 7 shows two theoretical farmer scenarios for variable-rate N application, conservative and risk taking, based on the number of N units to be distributed in the field. Usually, the farmer is reluctant to distribute fewer units than his experience would indicate are needed per hectare; therefore, a conservative scenario is necessary when the customary N units that are used in the area are distributed more in areas where higher yields are expected and less in areas with lower yield potential. With an average value of 240 kg N per hectare, Figure 7b,d represent the conservative scenario; the average per hectare units are maintained to be equal to the rates that are used without precision farming tools (in Figure 7b,d, max = 288 kg ha^−1^, med = 240 kg ha^−1^, and min = 192 kg ha^−1^). The significant savings occur in the risk-taking scenario, in which a producer decides to apply an average value of 240 kg N ha^−1^ in areas with the greatest potential and reduce the amount in areas with lower potential (in Figure 7a, max = 240 kg ha^−1^, med = 220 kg ha^−1^, and min = 200 kg ha^−1^; and Figure 7c, max = 240 kg ha^−1^, med = 225 kg ha^−1^, and min = 210 kg ha^−1^). The risk-taking strategy for variable-rate application of N reduce costs by approximately 20%. The risk taking strategy can be improved, for example, in cases in which the soil is the limitation to achieving higher yields.

All of maps shown in Figure 7 were generated on an appropriate grid (12 m × 12 m) using field positions from a GNSS receiver and a map of the desired application rate; the input rate changed as the spreader moved through the field. The N VRA machine should exhibit the highest real-time accuracy, but we observe that the mechanical nature of the application machine requires consideration of its limitations in the following areas: the working width (e.g., 12 m), minimum dose, maximum dose and time actuator settings. Another possible source of error is related to the characteristics of the applied material, e.g., the density and fluidity, which can change throughout the day; consequently, it is very important that the on-board equipment is well calibrated.

## 4. Conclusions

This ongoing research seeks new and improved tools to contribute to decision-making about the correct amount of N fertilizer to apply to winter wheat fields in a Mediterranean environment in a particular year. Until now, the adoption of VRA N management by producers has been very low, despite the potential economic and environmental benefits of this practice. Our major contributions are as follows:

- The average percentage error of yield monitoring for detecting the actual mass flow rate was −3.1% with a standard deviation of 4.2%. This monitoring enabled an assessment of the relationship between the yield data and NDVI measurements (r^2^ = 0.64 and 0.72) for fields 1 and 2.
- An assessment of the relationship between the wheat leaf N content and NDVI measurements from optical sensor values revealed coefficients of determination of greater than 0.9 when measured with the sensor.
- An appropriate and inexpensive portable hand-held optical sensor (GreenSeeker^®^, Trimble Navigation Ltd., Sunnyvale, CA, USA) could satisfactorily help operators predict and generate a map of N application recommendations for fields. Wheat canopy greenness may not always be the result of a certain N content (e.g., available water or temperature may also affect the greenness). If the greenness is not related to the N content, then N inputs are based on an erroneous indicator.
- N recommendation maps were developed, and accurate N recommendations for sub-regions of fields were produced. The recommended N maps based on this technique may help operators use accurate and efficient application rates from year to year.
